# Fine-Tuned Reactivity of *N*-Containing Naphthol Analogues †

**DOI:** 10.3390/ijms232012329

**Published:** 2022-10-15

**Authors:** Oszkár Csuvik, Petra Barta, Antal Csámpai, István Szatmári

**Affiliations:** 1 Institute of Pharmaceutical Chemistry, University of Szeged, Eötvös u. 6, H-6720 Szeged, Hungary; 2 Department of Organic Chemistry, Eötvös Loránd University, P.O. Box 32, H-1518 Budapest, Hungary; 3 Stereochemistry Research Group, Eötvös Loránd Research Network, University of Szeged, Eötvös u. 6, H-6720 Szeged, Hungary

**Keywords:** *ortho*-quinone methide, Mannich reaction, hydroxyquinoline, fine-tuned reactivity, reaction mechanism, DFT modelling, NMR structure elucidation

## Abstract

6-Hydroxyquinoline and 3-hydroxyisoquinoline as *N*-containing naphthol analogues were tested in modified Mannich reactions (*m*Mr’s). In the case of 6-hydroxyquinoline, the outcomes of the attempted Mannich reactions were strongly influenced by the amine components. Aminoalkylation of this substrate with reagents 1-naphthaldehyde and *N*-benzylmethylamine led to the isolation of a diol regarded as a stabilised water adduct of an *ortho*-quinone methide (*o*-QM), of which formation can be ascribed to the presence of a hydroxide ion in a relatively higher concentration generated by the bulky and basic amine component with decreased nucleophilicity. The classical Mannich base was isolated as a single product when the amine component was replaced for morpholine, featuring nucleophilicity rather than basic character under the applied reaction conditions. Starting from the isomer substrate 3-hydroxyisoquinoline, independently on the nucleophile (methanol or morpholine) besides the formation of the classical Mannich base, the nucleophilic attack at position one of the heterocyclic substrate was also observed. The DFT analysis of the acceptor molecular orbitals of the potential electrophilic components and the thermodynamics of the assumed-possible transformations demonstrated that this regioselective addition is a feasible process on the investigated heterocyclic skeleton. DFT modelling studies also suggest that besides the steric bulk, the orbital-controlled electronic properties of the aryl group, originating from the aldehyde components, have a strong influence on the ratios and the NMR-monitored interconversions of the C-1-substituted 3-hydroxyisoquinolines and the classical Mannich bases formed in multistep reaction sequences. On the basis of the DFT analysis of the thermodynamics of alternative pathways, a reaction mechanism was proposed for the rationalization of these characteristic substrate-controlled interconversions.

## 1. Introduction

The Mannich reaction is a widely used multicomponent reaction, providing a carbon–carbon bond formation under relatively mild reaction conditions [1,2]. According to the original reaction, a C–H acid, formaldehyde and a secondary amine were applied. In the modified Mannich reaction (*m*Mr), the C–H acid is replaced with an electron-rich aromatic compound, such as 1- or 2-naphthol [3,4], quinolinol or isoquinolinol [3,5], and, in a similar manner, formaldehyde with benzaldehyde. The formation of the Mannich product may be approached in two ways. The first is the nucleophilic addition of an electron-rich aromatic compound to the Schiff base [4], while the other assumes, first, the formation of an *ortho*-quinone methide (*o*-QM) that will be stabilised by reacting it with an amine component as a nucleophilic agent [6].

Quinolinols and isoquinolinols have a wide range of biological activity. 3-Hydroxyquinolines are cytotoxic [7], and 3-hydroxyisoquinolines are matrix metalloproteinase inhibitors [8]. Meanwhile, 6- and 7-hydroxyquinolines are anti-hepatitis B virus agents [9,10]. Moreover, 6-hydroxyisoquinolines are Na^+^, K^+^-ATPase inhibitors [11], and 7-hydroxyisoquinolines are selective kappa-opioid receptor antagonists [12].

**Figure 1:**
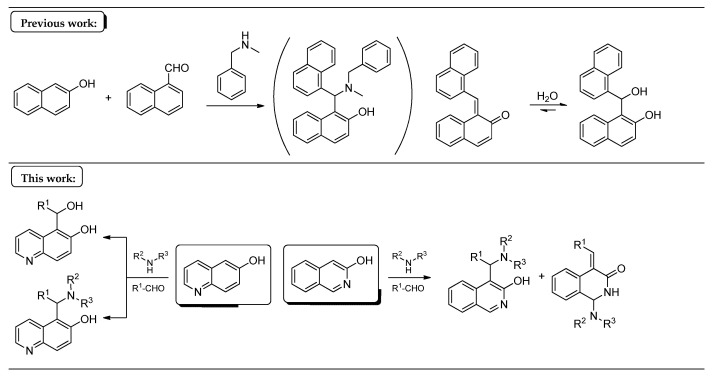
The summary of the present work with the previous findings.

In our previous study [13], 2-naphthol, 2-naphthaldehyde and *N-*benzylmethylamine were supposed to follow the mechanism of the *m*Mr’s. In contrast, the unexpected formation of 1-(hydroxy(naphthalen-1-yl)methyl)naphthalen-2-ol (a formal hydrate of an *o*-QM) was observed, instead of the classical Mannich base (Figure 1). Our aim in this work was to investigate the scope and limitations of the latter reaction (the stabilised benzylidene derivative vs. the Mannich base) starting from 6-hydroxyquinoline and 3-hydroxyisoquinoline as *N*-containing 2-naphthol substrates.

## 2. Results and Discussion

**Scheme 1:**
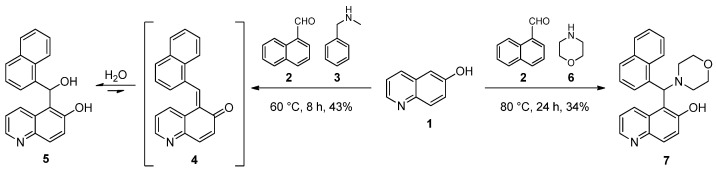
Formation of *ortho*-quinone methide (**4**) and its stabilisation to hydrate (**5**) and aminoalkylation of 6-hydroxyquinoline (**1**).

In our first experiments, 6-hydroxyquinoline (**1**) was reacted with 1-naphthaldehyde (**2**) and *N*-benzylmethylamine (**3**) under neat conditions (Scheme 1). Of the tested temperatures (60 °C, 80 °C and 100 °C), 60 °C was found to be optimal, which was provided by classical heating (an oil bath). After 8 h, in the course of the workup of the reaction mixture, the crude product was subjected to column chromatography. Subsequent crystallisation from methanol gave a mixture with hydrate **5** as the main component, which is the stabilised form of *ortho*-quinone methide **4** as disclosed by combined NMR methods. An analogous product was isolated in our previous work when 2-naphthol was attempted to be aminoalkylated with *N*-benzylmethylamine in the presence of 1-naphthaldehyde [12]. In order to evaluate the role of the amines, morpholine (**6**) as a secondary cyclic amine was reacted with 6-hydroxyquinoline and 1-naphthaldehyde under conditions that were previously optimised (Scheme 1). Thin-layer chromatography (TLC) confirmed the formation of a single product, which was then isolated by crystallisation. The NMR spectra provided evidence that the use of morpholine as an amine component led to the formation of the classical Mannich product **7**. The differences between the reaction pathways can be explained in terms of the different steric demands and the basicity of the applied amines (pK_b_ = 4.25 and 5.64 for **3** and **6**, respectively). Since **3** is bulkier and at least tenfold more basic than **6**, it can be more prone to activate water molecules rather than to attack as a nucleophile on the potential electrophilic species present in the reaction mixture.

**Scheme 2:**
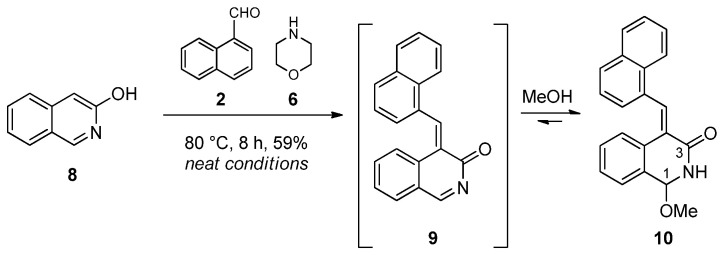
Selective substitution of 3-hydroxyisoquinoline (**8**).

In order to explore further structure–reactivity relationships, 3-hydoxyisoquinoline (**8**), a *N*-containing electron-rich aromatic substrate, was selected for the subsequent studies. Accordingly, 3-hydroxyisoquinoline (**8**), 1-naphthaldehyde (**2**) and *N*-benzylmethylamine (**3**) were reacted under neat conditions. After 30 minutes of reaction time at 60 °C, the prepared TLC was multi-spotted, and no well-defined component could be isolated from the reaction mixture. Thereafter, *N*-benzylmethylamine was switched to morpholine. A prolonged reaction time (6 h) at 80 °C was necessary for the appearance of a few new spots on the TLC. By means of column chromatography, using EtOAc:MeOH (20:1) as an eluent, we isolated methoxy-substituted lactam **10** as a distinct compound. In the ^1^H NMR spectrum of **10**, the typical singlet of 3H intensity at 3.35 ppm and the amide-type NH doublet at 9.25 ppm along with ^1^H-^13^C-HMBC connectivity between signal pairs OCH_3_/C-1, H-1/OCH_3_, H-1/C-3, NH/C-3 and NH/C-4 unambiguously proved its structure. The formation of this product can be explained via the formation of the *ortho*-quinone methide (**9**), which reacted at the most electrophilic *N*-acylimine site with methanol present in the solvent mixture used in the chromatographic purification (Scheme 2).

To investigate the influence of the arylidene moiety on the apparently unexpected nucleophilic attack of MeOH at position 1, 3-hydroxyisoquinoline (**8**) and methanol were stirred at 80 °C. Since, even after a prolonged reaction time (20 h), TLC indicated only the presence of the starting compounds, the reaction was repeated at 100 °C and 120 °C. Because mixing **8** with MeOH at a higher temperature resulted again in the presence of the unchanged starting compounds, we focused our attention on the evaluation of the effects of stronger nucleophiles, such as *N*-benzylmethylamine and morpholine, on the outcome of the reactions. First, *N*-benzylmethylamine (**3**) and 3-hydroxyisoquinoline (**8**) were reacted at 80 °C under neat conditions. Surprisingly, after 12 h of reaction time, TLC showed the formation of a multicomponent mixture.

**Scheme 3:**
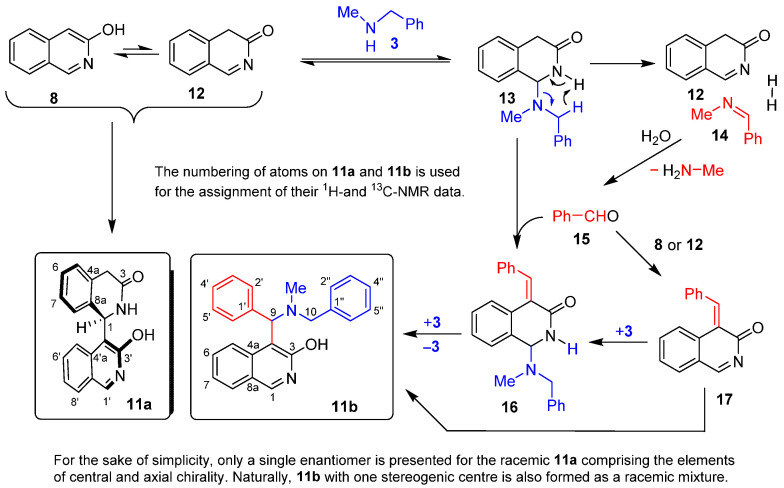
Unexpected transformations of 3-hydroxyisoquinoline (**8**) in the presence of *N*-benzylmethylamine (**3**).

After purification by column chromatography, two components with characteristic compositions (**11a** and **11b**: Scheme 3) could be isolated and identified by combined NMR methods, including the use of highly diagnostic ^1^H-^13^C-HMBC. The apparently intriguing transformations can be rationalised by the equilibrium formation of tautomer **12**, the key intermediate of the subsequent transformations. On one hand, the nucleophilic attack of tautomer **8** on the highly-electrophilic C-1 centre of this activated *N*-acyl-imine results in the formation of the racemic mixture of the chiral dimer **11a**. On the other hand, upon nucleophilic attack of **3**, tautomer **12** is converted into adduct **13**. The latter undergoes a synchronous imine- and dihydrogen-forming fragmentation, leading to Schiff base **14** with the simultaneous regeneration of **12**. Hydrolysis of **14** gives benzaldehyde **15** and methylamine. Upon condensation with **15,** saturated aminal adduct **13** is transformed into **16**. This enone-containing aminal intermediate then undergoes sequential addition and elimination of **3,** eventually affording **11b**, the other isolated aromatic product. Finally, either of the two equilibrating tautomers of the heterocyclic precursor (**8** or **12**) might also react with **15** to construct enone **17**. The *Aza*-Michael addition of the latter with **3** also leads to **11b**.

**Scheme 4:**
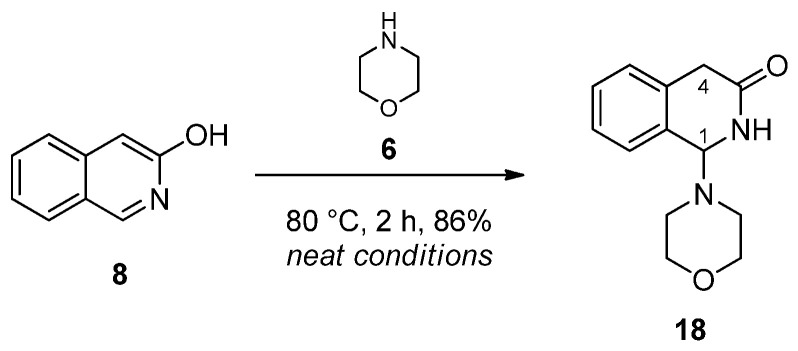
Reaction of 3-hydroxyisoquinoline (**8**) and morpholine (**6**).

Since *N*-benzylmethylamine was expected to undergo uncontrolled decomposition, leading to a wide range of side products, morpholine was applied in our subsequent experiments. This stable secondary cyclic amine was stirred with 3-hydroxyisoquinoline at 80 °C under neat conditions. After 2 h, the spot indicating 3-hydroxyisoquinoline disappeared on a TLC; therefore, the mixture was worked up by neutral column chromatography to produce aminal **18** as a single isolated product (Scheme 4). In its ^1^H-NMR spectrum, the separated AB signals (*J* = 20 Hz) of the diastereotopic H-4 protons discernible at 3.37 ppm and 3.64 ppm indicated its lactame-type skeletal structure with a stereogenic centre at position 1. This structure was also supported by the chemical shift of C-1 (171.4 ppm) and by HMBC cross peaks revealing correlations between ^1^H/^13^C coupled pairs H-4A/C-1, H-4B/C-1, H-1/C-1 and NH/C-1 (Scheme 4).

**Figure 2:**
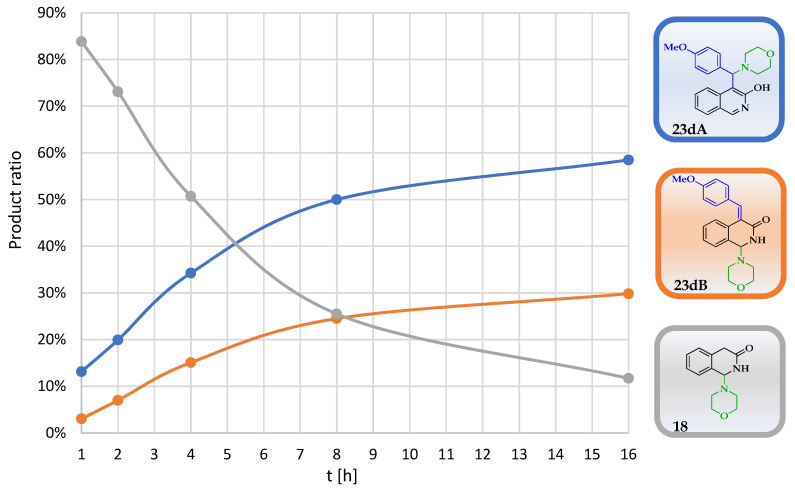
The time-dependent ratios of the morpholino-hydroxyquinoline adduct **18,** the Mannich product **23dA** and the 4-methoxybenzylidene derivative **23dB** as determined by ^1^H NMR.

In order to unambiguously clarify the role of the amine and the aldehyde in this reaction and to avoid the rather unpredictable reactivity and decomposition of *N*-benzylmethylamine, 3-hydroxyisoquinoline (**8**) was reacted with morpholine (**6**) in the presence of different aromatic aldehydes under neat conditions. Since the incorporation of the methoxy group in the case of **10** was also undesirable, the composition of the crude reaction mixtures was analysed using ^1^H NMR without any further purification. In accord with our previous findings with morpholine as the amine nucleophile, in addition to **18**, we detected two types of products (**23A** and **23B**) in a time-dependent manner. Thus, the crude products were analysed at five different reaction times (1 h, 2 h, 4 h, 8 h and 16 h). In general, the first-appearing NMR signals can unambiguously be assigned to the protons of **18**. In the progress of the experiments, the ratio of **18** was gradually decreased, while the ratios of the other products were increased. As a representative example, the transformation of **23d** is demonstrated in Figure 2.

The ratio of the regioisomers was obtained as the relative intensity of the diagnostic proton signals of particular components. Namely, a singlet in the range of 5.3–5.5 ppm is characteristic for Mannich adducts type **23A**, and a doublet in the range of 5.0–5.1 ppm is diagnostic for enone aminals of type **23B** (Table 1). The relative amount of **18** could also be calculated by the intensity of the characteristic proton signals at 5.03 ppm and 8.43 ppm.

**Figure 3:**
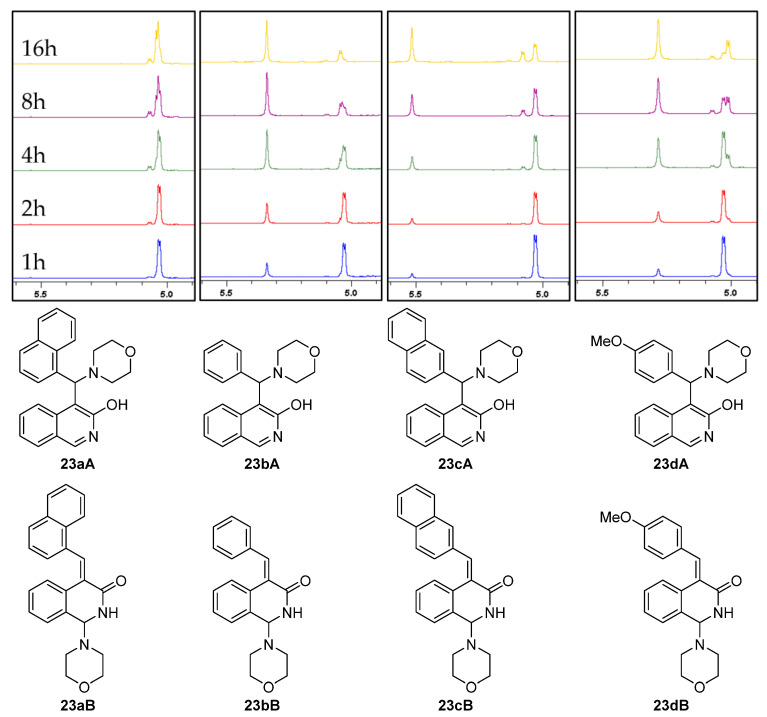
Structures and diagnostic ^1^H NMR signals of Mannich products (type **23A**) and arylidene derivatives (type **23B**).

The ^1^H NMR analyses revealed the parallel formation of the classical Mannich product (**23b**–**dA**) and the corresponding arylidene derivative (**23b**–**dB**) when benzaldehyde (**15**), 2-naphthaldehyde (**19**) and 4-methoxybenzaldehyde (**20**) were applied as the carbonyl component (Figure 3). However, independently of the reaction time, when 1-naphthaldehyde (**2**) was used as a coupling partner, the reactions led to the formation of the arylidene derivative **23aB**, which could be isolated as a single product.

It must be pointed out that enones **23b**–**dB** were slowly transformed into the appropriate Mannich-type product **23b**–**dA** as highlighted by the ^1^H NMR spectra of the reaction mixtures that were registered at different times (Figure 3).

**Scheme 5:**
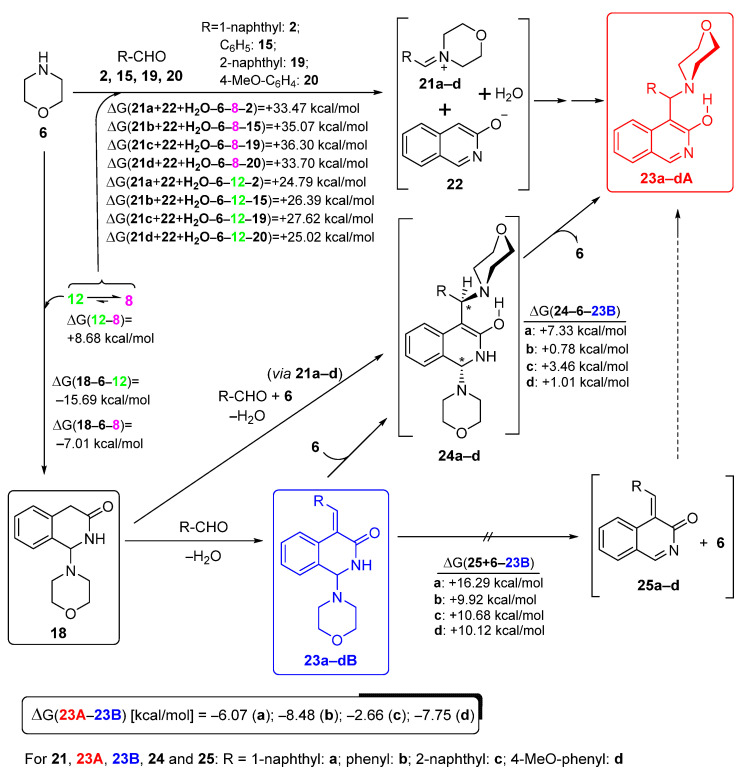
Mechanism of the formation of Mannich and benzylidene products (types 23A and 23B, respectively) proposed on the basis of the aryl group-dependent relative thermodynamics of the assumed transformations obtained using DFT modelling.

**Figure 4:**
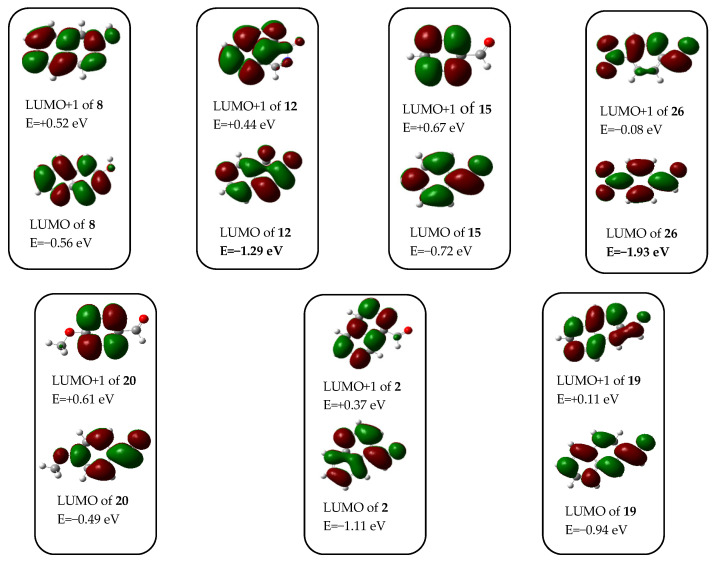
Acceptor orbitals (LUMO and LUMO + 1) of the electrophilic components involved in the Mannich-type reactions obtained using MO analysis of the optimized structures.

In order to rationalise all the aforementioned aryl-group-dependent experimental findings as well as the exceptional reactivity of 4-nitrobenzaldehyde **26** manifested in its analogue transformation to attempt to obtain the Mannich-type product (discussed later), we undertook comparative DFT modelling studies on the aryl-group-dependent progress of the formation and possible interconversion of **18** and compound types **23A** and **23B**. We hypothesised that these transformations might take place along the pathways outlined on Scheme 5. The feasibility of this assumption was assessed by the relative energetics of particular elementary steps and by the analysis of the acceptor orbitals (LUMO and LUMO + 1) of the possible electrophilic species involved in these Mannich-type and related conversions (Figure 4).

**Scheme 6:**
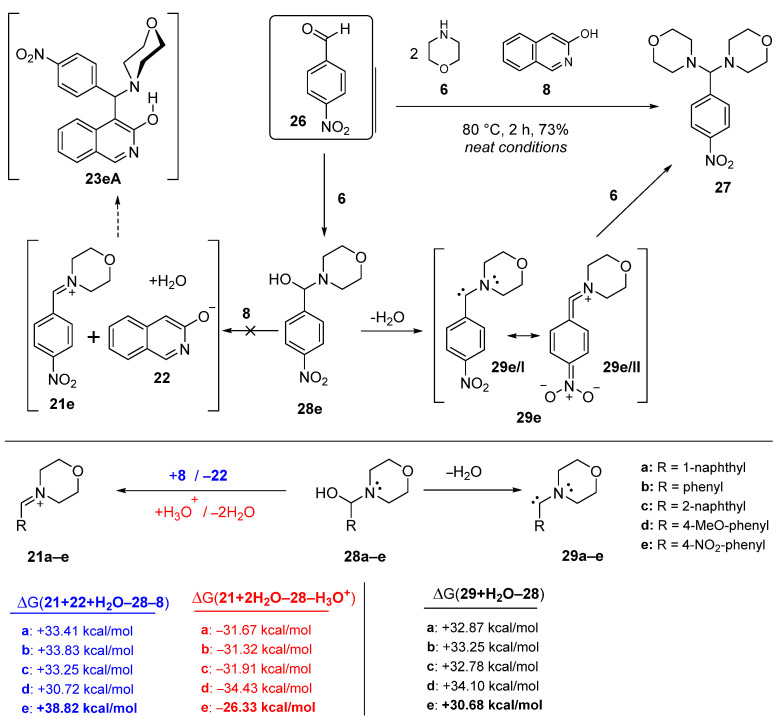
DFT-supported rationale of the unique reactivity of 4-nitrobenzaldehyde (**26**) manifested in its attempted Mannich condensation with morpholine (**6**) and 3-hydroxyisoquinoline (**8**) conducted under neat conditions.

First, the classical Mannich products were expected to be formed through an ion pair incorporating iminium cations **21a**–**d** and a heterocyclic anion **22** generated by the nucleophilic attack of **6** on the aldehyde component followed by the 3-hydroxyisoquinoline-promoted elimination of water. However, on the basis of the assumed relatively small amount of the ion pair **21b**–**d/22** [cf., the changes in Gibbs free energy (ΔG) accompanying their formation by implication of either tautomers **8** or **12**, as presented on Scheme 6] and the dominance of **18** in the reaction mixtures obtained after 1 h, the share of these pathways was negligible in the construction of compounds type **21A**. On the other hand, the rapid and dominant primary formation of **18** could be ascribed to the pronounced electrophilicity of **12**, mainly ascribed to its low-energy LUMO, with a significant share on the C-1 atom. Accordingly, except for 4-nitrobenzaldehyde **26**, the other arylaldehyde components had markedly higher LUMO energy relative to that of **12**. This oxo-tautomer could be regarded as the main precursor of **18**, even though the calculated ΔG value suggested that its tautomer **8** must be present in the reaction mixture in a substantially higher concentration. In the subsequent steps, **18** could have undergone condensation with the appropriate aldehyde to construct arylidenes **23B**, or, with the involvement of **6** generating iminium ions **21a**–**d**, it could have been converted into *bis*-morpholine intermediates type **24**. Morpholine elimination from the aminal residue of **24** gave the corresponding Mannich product (**23A**). It is of note that due to the thermodynamically unfavoured formation of **21a**–**d** under slightly acidic conditions (cf., data presented in Scheme 6), this condensation–elimination sequence can be considered as a less feasible pathway. Since the relative energetic data calculated for all investigated isomer pairs **23A**/**23B** [(ΔG(**23B**-**23A**), see Scheme 5] suggested that **23a**–**dA** are formed under thermodynamic control, the isomerisation of **23B** into **23A** can also be taken into account as a realistic process taking place under the applied conditions. In this regard, the calculated energetic data [(ΔG(**25**+**6**-**23B**)] practically rule out the elimination–addition sequence **23B**→**25**→**23A** as a realistic pathway because the competitive addition–elimination sequence proceeding via *bis*-morpholine intermediates **24** seems to be more feasible as indicated again by the relative energetics calculated for the critical addition step [(ΔG(**24**-**6**-**23B**)].

In this context, the relatively increased reluctance of **23aB** to undergo isomerisation into **23aA** seems to be the consequence of the pronounced endoergic addition step **23aB** + **6**→**24a**, which might be associated not only with its enhanced electron-donating character, but also with the steric bulk of the 1-naphthyl group.

The reaction of **8** and **6** with *p*-nitrobenzaldehyde (**26**, [14,15,16]) was an exceptional case, because neither the expected Mannich product **23eA** nor the appropriate benzylidene product **23eB** could be isolated. Instead, 4,4′-((4-nitrophenyl)methylene)dimorpholine (**27**) was formed as the main product (Scheme 6). This unique conversion of **26,** proceeding without the implication of equilibrating heterocyclic tautomers (**8** and **12**), can be interpreted by its LUMO energy being markedly lower than that of **12** (Figure 4). Furthermore, the relatively low-energy LUMO+1, also available to nucleophilic attack with significant share on the carbonyl group, further attenuates the electrophilicity of this reactive aldehyde. On the other hand, the tendency in the calculated energetic data—in accord with the general qualitative expectations—clearly shows that 3-hydroxyisoquinoline-promoted generation of the ion pair **21e**/**22** is a less favoured process, than the formation of the ion pairs **21a**–**d/22** containing a positively charged nitrogen atom without a strong electron-withdrawing substituent on the aryl group. It must be pointed out here that the readiness of iminium generation is strongly dependent on the proton source as shown by the energetic data obtained by replacing **8** with a hydroxonium ion in the calculations. The extreme difference between the two series of energetic data, otherwise both demonstrating the same tendency in the function of the electronic character of the aryl groups, can be considered as a strong indication of the dramatic dependence of the thermochemistry of ion-pair formation on a number of factors that might influence the stability of the charged particles, including, e.g., coulombic interaction, solvation and other intermolecular contacts. Avoiding unrealistically demanding computations, except for the estimated polarity of the reaction mixture (cf., description of the computational model in the “Materials and Methods” section), the aforementioned factors were neglected in the course of our DFT modelling studies.

Finally, we proposed a mechanism for the formation of **27**, accounting for taking place without the implication of **8**. Thus, according to our assumption, reductive elimination of the elements of the water molecule from the selectively generated adduct **28e** gives carbene **29e** stabilised by the 4-nitrophenyl group as represented by resonance hybrids **29e/I** and **29e/II**, which then reacts with **6** in an oxidative addition step affording **27** as the single isolable product (Scheme 6). In this sequence, carbene formation is the critical step with indispensable assistance of the nitrophenyl group. This view was supported by the comparison of the relative energetics of water elimination from adducts **28a**–**e** (Scheme 6). The calculated data [(ΔG(**29**+H_2_O-**28**)] unambiguously indicated that the process **28e**→**29e**+H_2_O is significantly less endoergic, and, consequently, it is more feasible than the analogous reaction steps **28a**–**d**→**29a**–**d**+H_2_O. It must be emphasised again, that it is the tendency of the relative energetics that must be regarded to have diagnostic value in the assessment of the substituent effect, as solvation of the involved species along with other stabilizing and destabilizing interactions—which were not modelled in the calculations—are also expected to determine the real thermochemistry of the studied conversions. It is also of note that carbene **29e** reacts with **6** rather than with **8**. This substrate selectivity might be associated with the enhanced nucleophilicity of **6** compared to the neutral **8** of which the deprotonation-generating ion pair **21e**/**22** with the highly reactive anion **22**, a real competitor of **6**, is suppressed by the nitrophenyl group as discussed above. 

## 3. Materials and Methods

The ^1^H and ^13^C NMR spectra were recorded in DMSO and D_2_O solutions in 5 mm tubes, at room temperature, with a Bruker spectrometer at 500 (^1^H) and 125 (^13^C) MHz, with the deuterium signal of the solvent as the lock and TMS as the internal standard.

Melting points were determined on a Hinotek X-4 melting point apparatus. Elemental analyses were performed with a Perkin-Elmer 2400 CHNS elemental analyser. Merck Kieselgel 60F254 plates were used for TLC.

In the course of DFT calculations, the optimisation and frequency calculations on molecular structures were carried out with M06-2X global functional [17] using 6-31 G(d,p) basis set [18] supplemented by IEFPCM solvent model [19]. For these computations, dielectric constant (ε) was set to 10 (an arbitrarily modified value of 7.3 and an accessible parameter for morpholine), approximately representing the polarity of the modelled reaction mixtures composed of 3:1:1 molar ratio of morpholine, 3-hydroxyisoquinoline and the corresponding arylaldehyde. The energetic profile of the transformations was given by the changes in Gibbs free energy (ΔG). The free energy values of the optimised structures were obtained by correcting the computed total energy with zero-point vibrational energy (ZPE) and thermal corrections calculated at the same level of theory.

All calculations were carried out using the Gaussian 09 software (Gaussian Incorporation, Pittsburgh, PA, USA) package [20]. The optimised structures are available from the authors.

The NMR spectra of **11a** and **11b** are described in more detailed because of its unexpected and complex structure.

### 3.1. Synthesis of 5-(hydroxy(naphthalen-1-yl)methyl)quinolin-6-ol (***5***)

6-Hydroxyquinoline (50 mg, 0.35 mmol), 1-naphtaldehyde (54 mg, 0.35 mmol) and *N*-benzylmethylamine (42 mg, 0.35 mmol) were placed in a 10 mL reaction vial and heated in an oil bath at 60 °C for 8 h under neat conditions. The crude mixture was purified by column chromatography (EtOAc: MeOH, 20:1) and crystallised from MeOH (5 mL). Yellowish solid; yield: 43% (45 mg); m.p.: 84–87 °C. ^1^H NMR (DMSO-d_6_): δ = 6.50 (1H, s); 7.26 (1H, dd, *J* = 8.1 Hz and 4.5 Hz); 7.37 (1H, br ~t, *J* ~ 8 Hz); 7.43–7.47 (2H, m); 7.60 (1H, t, *J* = 7.8 Hz); 7.84–7.89 (4H, m); 7.97 (1H, d, *J* = 7.8 Hz); 8.18 (1H, dd, *J* = 8.1 Hz and 1.8 Hz); 8.56 (1H, dd, *J* = 4.5 Hz and 1.9 Hz). ^13^C NMR (DMSO-d_6_): δ = 63.1; 117.1; 117.3; 121.7; 125.9; 126.5; 127.4; 127.9; 129.1; 130.2; 129.2; 130.8; 132.5; 134.1; 136.2; 144.0; 147.1; 156.3. (Figures S1–S3).

### 3.2. Synthesis of 5-(morpholino(naphthalen-1-yl)methyl)quinoline-6-ol (***7***)

6-Hydroxyisoquinoline (250 mg, 1.72 mmol), 1-naphtaldehyde (268 mg, 1.72 mmol) and morpholine (180 mg, 2.06 mmol) were placed in a 50 mL round-bottom flask and stirred at 80 °C for 24 h. The crude mixture was crystallised from EtOAc (7 mL) and recrystallised from *i*Pr_2_O (8 mL). Beige solid; yield: 34% (194 mg); m.p.: 189–192 °C. ^1^H NMR (DMSO-d_6_): δ = 3.59–3.68 (4H, m); 6.37 (1H, s); 7.21–7.26 (1H, m); 7.39–7.46 (2H, m); 7.60 (1H, t, *J* = 7.8 Hz); 7.70–7.78 (2H, m); 7.82–7.88 (2H, m); 7.97 (1H, d, *J* = 8.4 Hz); 8.14 (1H, brs); 8.56 (1H, s); 8.92 (1H, s). ^13^C NMR (DMSO-d_6_): δ = 63.7; 66.8; 116.6; 121.7; 123.6; 123.8; 126.3; 126.4; 127.3; 127.5; 127.9; 129.2; 129.6; 130.8; 132.4; 133.9; 143.9; 147.1. (Figures S4 and S5).

### 3.3. Synthesis of (E)-1-methoxy-4-(naphthalen-1-ylmethylene)-1,2-dihydroisoquinolin-3(4H)-one (***10***)

3-Hydroxyisoquinoline (50 mg, 0.34 mmol), 1-naphtaldehyde (53 mg, 0.34 mmol) and *N*-benzylmethylamine (41 mg, 0.34 mmol) were heated under solvent-free conditions in an oil bath at 80 °C for 8 h. The mixture was purified by column chromatography (EtOAc:MeOH, 20:1) and crystallised from MeOH (10 mL). Pale brown solid; yield: 59% (64 mg); m.p.: 187–190 °C. ^1^H NMR (DMSO-d_6_): δ = 3.36 (3H, s); 5.42 (1H, d, *J* = 4.5 Hz); 6.75 (1H, d, *J* = 8.1 Hz); 6.91 (1H, t, *J* = 7.3 Hz); 7.20 (1H, t, *J* = 7.7 Hz); 7.40 (1H, d, *J* = 7.3 Hz); 7.43–7.59 (4H, m); 7.90–8.01 (3H, m); 8.13 (1H, s); 9.28 (1H, d, *J* = 4.5 Hz). ^13^C NMR (DMSO-d_6_): δ = 54.1; 83.0; 124.7; 126.2; 126.6; 126.9; 127.2; 127.5; 128.2; 128.2; 129.1; 129.2; 130.9; 131.1; 131.3; 133.7; 133.8; 134.0; 167.6. (Figures S6 and S7).

### 3.4. Reaction of 3-Hydroxyisoquinoline with N-Benzylmethylamine

The mixture of *N*-benzylmethylamine (168 mg, 1.39 mmol) and 3-hydroxyisoquinoline (50 mg, 0.35 mmol) was placed in a 10 mL reaction vial and heated in an oil bath at 80 °C for 12 h. Dichloromethane (50 mL) and water (50 mL) were added to the mixture that was then extracted with 2% hydrochloric acid. After extraction, the organic fractions were collected, dried with anhydrous Na_2_SO_4_ and filtered, and the solvent was removed under reduced pressure. The residue was crystallised with *n*-hexane (15 mL) and recrystallised from *i*Pr_2_O (10 mL) to afford **11a**. The aqueous phase was alkalised with Na_2_CO_3_ and extracted with dichloromethane (2 × 50 mL). The organic fractions were collected, dried on Na_2_SO_4_, filtered and evaporated. The residue was purified by column chromatography (eluent EtOAc) to afford **11b**.

#### 3.4.1. 3′-Hydroxy-1,2-dihydro-[1,4′-biisoquinolin]-3(4H)-one (**11a**)

Yield: 13% (13 mg); m.p.: 181–184 °C. ^1^H NMR (DMSO-d_6_): δ = 3.67 (1H, d, *J* = 21.6 Hz, H-4A); 3,71 (1H, d, *J* = 21.6 Hz, H-4B); 6.53 (1H, br s, H-1); 6.65 (1H, br d, *J* = 7.8 Hz, H-8); 7.03 (1H, t, *J* = 7.8 Hz, H-7); 7.20 (1H, t, *J* = 7.8 Hz, H-6); 7.25–7.28 (2H, two overlapping m’s, H-5 and H-5′); 7.35 (1H, m, H-7′); 7.47 (1H, br m, H-6′); 7.96 (1H, d, *J* = 8.0 Hz, H-8′); 8.15 (1H, br s, H-2); 8.88 (1H, s, H-1′). ^13^C NMR (DMSO-d_6_): δ = 36.6 (C-4); 51.2 (C-1); 111.3 (C-4′); 123.0 (C-5′); 123.2 (C-7′); 123.7 (C-8′a); 125.4 (C-8); 127.1 (C-7); 127.3 (C-6); 128.0 (C-5); 129.3 (C-8′); 131.2 (C-6′); 132.8 (C-4a); 134.9 (C-8a); 137.6 C-4′a); 149.4 (C-1′); 159.3 (C-3′); 169.9 (C-3). (Figures S8–S10).

#### 3.4.2. 4-((Benzyl(methyl)amino)(phenyl)methyl)isoquinolin-3-ol (**11b**)

Yield: 15% (18 mg); m.p.: 153–156 °C. ^1^H NMR (DMSO-d_6_): δ = 2.09 (3H, s, NCH_3_); 3.58 (2H, br s, H-10); 5.47 (1H, s, H-9); 7.21 (1H, t, *J* = 7.8 Hz, H-7); 7.27–7.40 (9H, overlapping m’s, H-5, H-3′,5′, H-4′, and H-2″-6″); 7.65 (1H, t, *J* = 7.8 Hz, H-6), 7.71 (2H, d, *J* = 7.7 Hz, H-2′,6′); 7.94 (1H, d, *J* = 7.8 Hz, H-8); 8.88 (1H, s, H-1). ^13^C NMR (DMSO-d_6_): δ = 40.0 (NCH_3_); 60.0 (C-10); 68.6 (C-9); 110.7 (C-4); 124.0 (C-5); 128.1 (C-3′,5′); 128.3 (C-7); 128.4 (C-8a); 128.5 (C-4′); 128.6 (C-2′,6′); 129.0 (C-4″); 129.4 (C-3″,5″); 129.6 (C-2″,6″); 129.9 (C-8); 131.3 (C-6); 136.7 (C-4a); 138.1 (C-1″); 141.5 (C-1′); 151.0 (C-1); 159.5 (C-3). (Figures S11–S13).

### 3.5. Synthesis of 1-morpholino-1,2-dihydroisoquinolin-3(4H)-one (**18**)

3-Hydroxyisoquinoline (50 mg, 0.34 mmol) and morpholine (120 mg, 1.38 mmol) were placed in a 25 mL round-bottom flask and were stirred at 80 °C for 2 h under neat conditions in an oil bath. The crude mixture was crystallised with EtOAc (12 mL). Beige solid; yield: 86% (69 mg); m.p.: 166–170 °C. ^1^H NMR (DMSO-d_6_): δ = 2.40 (4H, t, *J* = 4.5 Hz); 3.37 (1H, d, *J* = 20.0 Hz); 3.46–3.55 (4H, m); 3.63 (1H, d, *J* = 20.0 Hz); 5.03 (1H, d, *J* = 3.5 Hz); 7.18 (1H, d, *J* = 7.1 Hz); 7.25–7.31 (2H, m); 7.36 (1H, d, *J* = 7.2 Hz); 8.44 (1H, d, *J* = 3.1 Hz). ^13^C NMR (DMSO-d_6_): δ = 36.3; 47.7; 66.6; 74.2; 126.7; 127.7; 128.3; 128.3; 132.0; 133.8; 171.4. (Figures S14 and S15).

### 3.6. Synthesis of Compounds ***23***

The mixture of 0.34 mmol aldehyde [1-naphthaldehyde (53 mg), 2-naphthaldehyde (53 mg), benzaldehyde (36 mg), 4-methoxybenzaldehyde (47 mg) or 4-nitrobenzaldehyde (52 mg)], morpholine (90 mg, 1.03 mmol) and 3-hydroxyisoquinoline (50 mg, 0.34 mmol) were placed in a 10 mL reaction vial and heated in an oil bath at 80 °C under neat conditions.

#### 3.6.1. (E)-1-Morpholino-4-(naphthalen-1-ylmethylene)-1,2-dihydroisoquinolin-3(4H)-one (**23aB**)

The crude mixture was purified by column chromatography (EtOAc:Aceton, 3:1) and crystallised from Et_2_O/EtOAc (10 mL). Beige solid; yield: 24% (31 mg); m.p.: 127–129 °C. ^1^H NMR (DMSO-d_6_): δ = 2.55–2.65 (4H, m); 3.56–3.66 (4H, m); 5.04 (1H, s); 6.72 (1H, d, *J* = 7.7 Hz); 6.87 (1H, t, *J* = 7.6 Hz); 7.19 (1H, t, *J* = 7.2 Hz); 7.38 (2H, t, *J* = 7.3 Hz); 7.47 (1H, t, *J* = 7.9 Hz); 7.53 (1H, t, *J* = 7.5 Hz); 7.58 (1H, t, *J* = 7.8 Hz); 7.94 (2H, d, *J* = 8.1 Hz); 8.00 (1H, d, *J* = 7.9 Hz); 8.12 (1H, s); 8.86 (1H, s). ^13^C NMR (DMSO-d_6_): δ = 48.0; 66.7; 73.2; 124.7; 126.1; 126.3; 127.0; 127.2; 127.6; 128.1; 129.0; 129.1; 129.1; 131.1; 131.3; 132.0; 133.0; 133.2; 133.8; 134.0; 167.5. (Figures S16 and S17).

#### 3.6.2. 4-(Morpholino(phenyl)methyl)isoquinolin-3-ol (**23bA**)

The crude mixture was purified by column chromatography (EtOAc:Aceton, 4:1, and DCM:Aceton, 19:1) and crystallised from Et_2_O (9 mL). Beige solid; yield: 17% (15 mg); m.p.: 165–168 °C. ^1^H NMR (DMSO-d_6_): δ = 2.38–2.48 (4H, m); 3,67 (4H, s); 5.34 (1H, s); 7.20 (1H, t, *J* = 7.4 Hz); 7.27–7.35 (3H, m); 7.58–7.65 (3H, m); 7.92 (1H, d, *J* = 8.3 Hz); 8.33 (1H, brs); 8.86 (1H,s). ^13^C NMR (DMSO-d_6_): δ = 29.5; 52.5; 66.6; 68.5; 123.8; 124.7; 128.2; 128.7; 129.1; 129.2; 131.2; 136.8; 159.3. (Figures S18 and S19).

## 4. Conclusions

In conclusion, 6-hydroxyquinoline and 3-hydroxyisoquinoline as *N*-containing naphthol analogues were tested in the *m*Mr. By the reaction of 6-hydroxyquinoline and 1-naphthaldehyde in the presence of *N*-benzylmethylamine, diol **5** was isolated. By replacing the amine compounds, the classical Mannich base formed as a single product. The difference between the products was interpreted on the basis of the different basicity of the starting amines. When 3-hydroxyisoquinoline was aminoalkylated in the presence of aromatic aldehydes, in addition to the classical Mannich base, the parallel formation of the C-1-substituted 3-hydroxyisoquinolines was observed. On the basis of comparative DFT modelling studies, it was concluded that, besides the steric bulk, the orbital-controlled electronic properties of the potential electrophilic components including aldehydes and, in particular, the carbonyl tautomer of 3-hydroxyisoquinoline (**12**) have a strong influence on the ratios and interconversions of the C-1-substituted 3-hydroxyisoquinolines and the classical Mannich bases. The unique reactivity of 4-nitrobenzaldehyde manifested under the same conditions was also rationalised by a distinct mechanism proposed on the basis of DFT modelling studies. The characteristic structure–reactivity relationships disclosed in this contribution along with the mechanisms we proposed for the intriguing transformations might be taken into account in the design of related multicomponent reactions targeted to construct valuable products with complex molecular architectures including, e.g., compounds of biological relevance.

## Figures and Tables

**Table 1:** The characteristic proton signals discernible in the ^1^H-NMR spectra of the reaction mixtures.

Reaction	Characteristic Protons of the Compounds		
**23a**	**18**	**23aA**	**23aB**
	5.03 ppm	–	5.04 ppm
**23b**	**18**	**23bA**	**23bB**
	5.03 ppm	5.34 ppm	5.05 ppm
**23c**	**18**	**23cA**	**23cB**
	5.03 ppm	5.51 ppm	5.09 ppm
**23d**	**18**	**23dA**	**23dB**
	5.03 ppm	5.28 ppm	5.02 ppm

## Data Availability

The data generated and analyzed during our research are not available in any public database or repository but will be shared by the corresponding author upon reasonable request.

## Supplementary Materials

The following supporting information can be downloaded at: https://www.mdpi.com/article/10.3390/ijms232012329/s1.
