# Supplementary material for: Fine-Tuned Reactivity of N-Containing Naphthol Analogues
Source: Int J Mol Sci. 2022 Oct 15;23(20):12329. doi: 10.3390/ijms232012329 (PMC9604367; doi:10.3390/ijms232012329)
Supplement: Supplementary file 1 [file ijms-23-12329-s001.zip › ijms-1936409-supplementary.pdf]

Fine-tuned reactivity of *N*-containing naphthol analogues

Oszkár Csuvik [1], Petra Barta [1], Antal Csámpai [2,*], and István Szatmári [1,3,*]

[1] Institute of Pharmaceutical Chemistry, University of Szeged, Eötvös u. 6, H-6720 Szeged, Hungary
[2] Department of Organic Chemistry, Eötvös Loránd University, P.O. Box 32, H-1518 Budapest, Hungary
[3] Stereochemistry Research Group, Eötvös Loránd Research Network, University of Szeged, Eötvös u. 6, H-6720 Szeged, Hungary
* Correspondence: antal.csampai@ttk.elte.hu (A.C.); szatmari.istvan@szte.hu (I.S.)

Contents

1. ^{1}H NMR, and ^{13}C NMR spectra of synthesised compounds……………………….2

5-(hydroxy(naphthalen-1-yl)methyl)quinolin-6-ol (5)

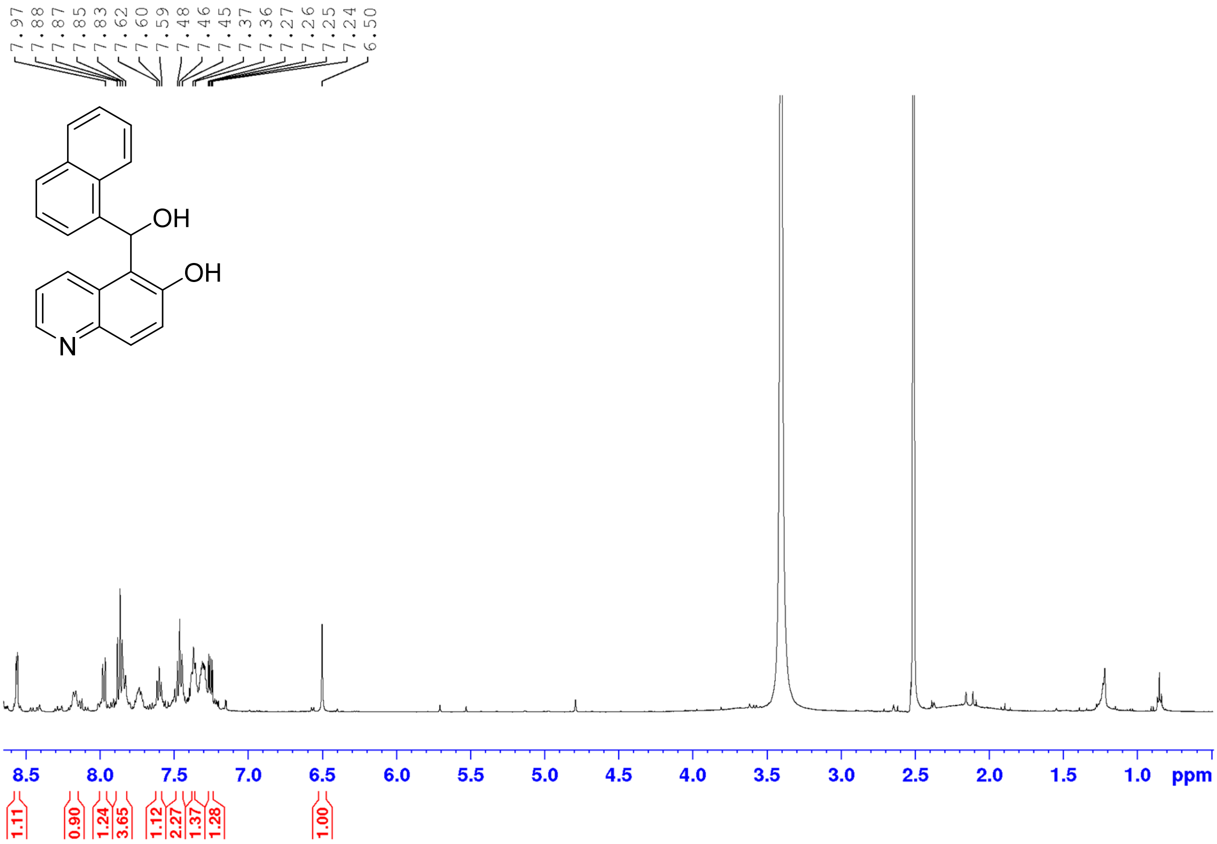

Figure S1. ^{1}H-NMR spectrum of **5**

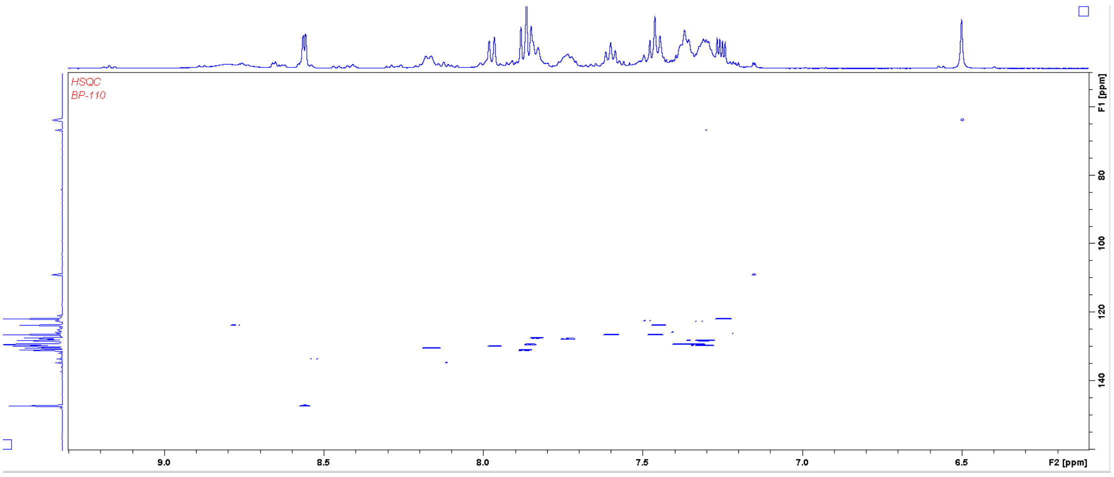

Figure S2. HSQC spectrum of **5**

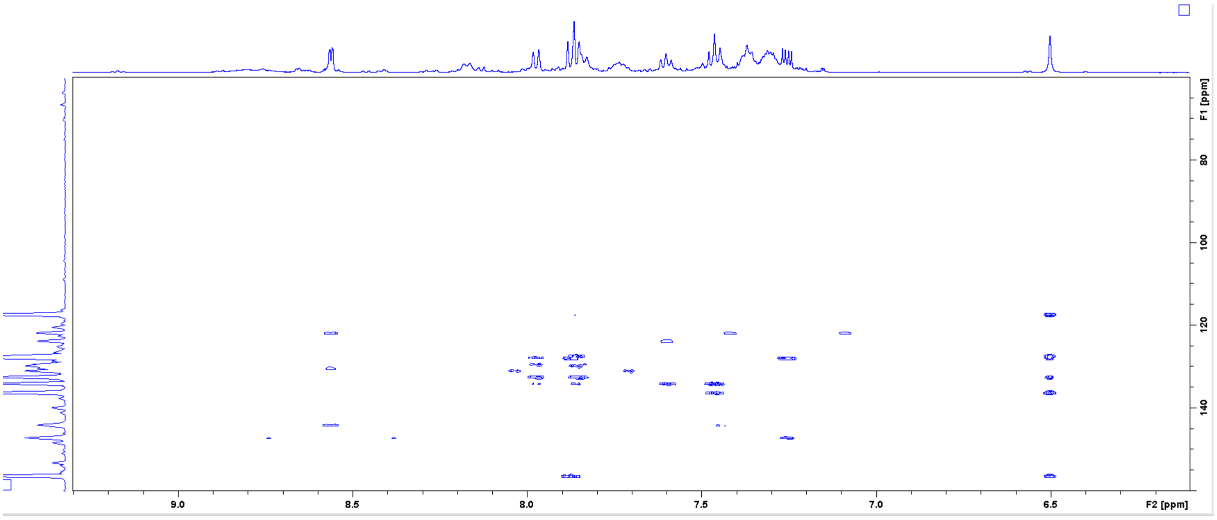

Figure S3. HMBC spectrum of **5**

5-(morpholino(naphthalen-1-yl)methyl)quinolin-6-ol (7)

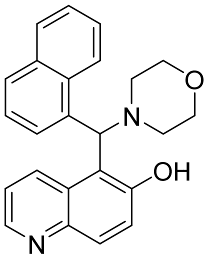

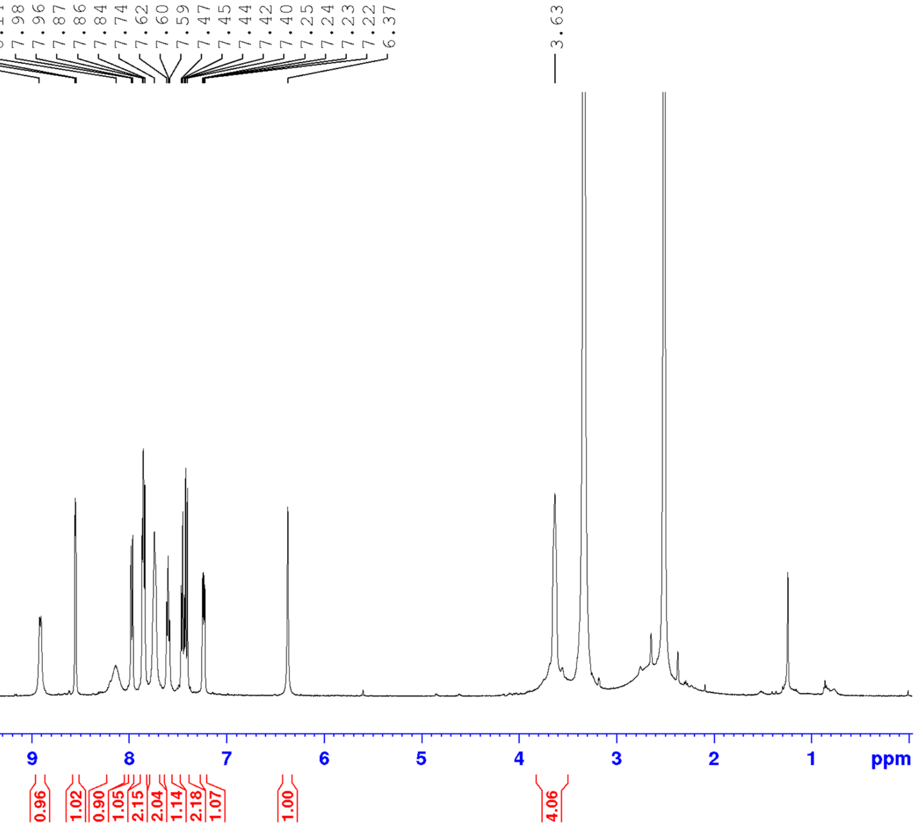

Figure S4. ^{1}H-NMR spectrum of **7**

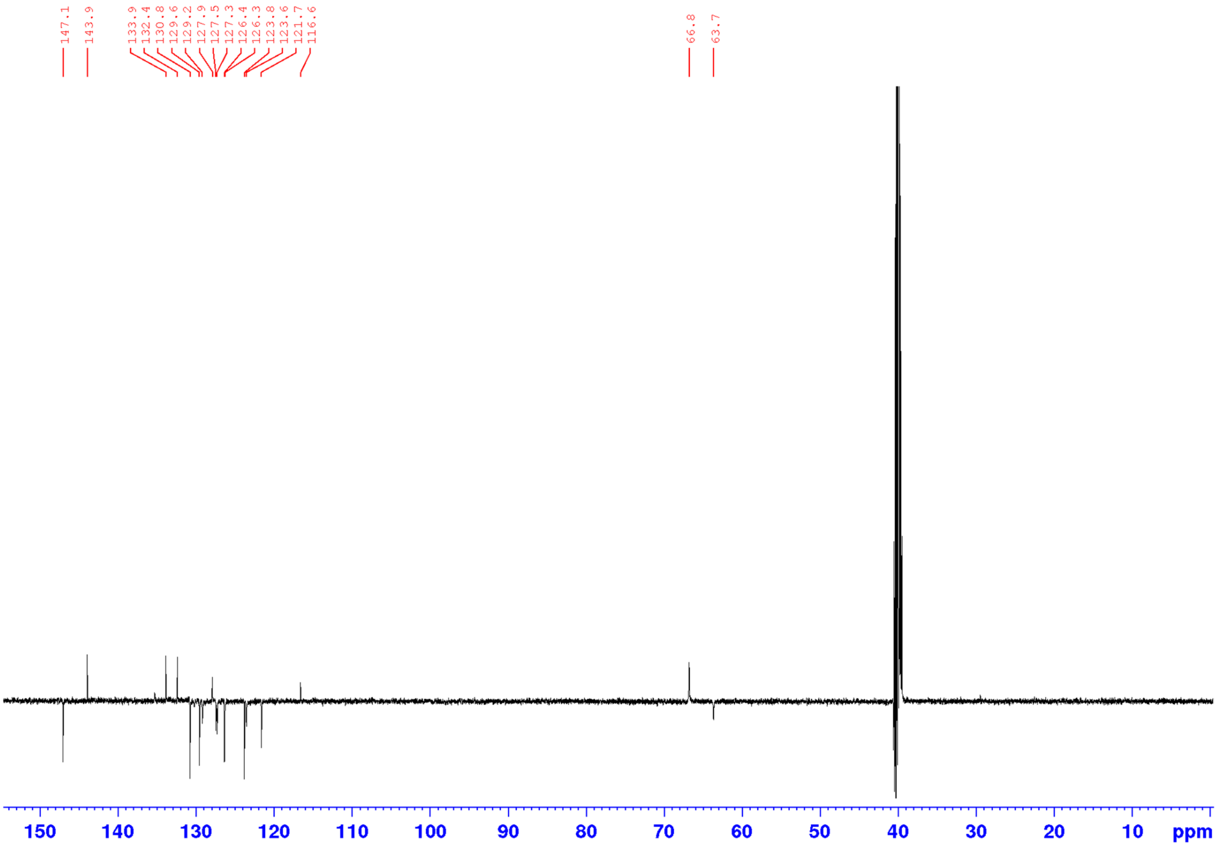

Figure S5. ^{13}C-NMR spectrum of **7**

1-methoxy-4-(naphthalen-1-ylmethylene)-1,2-dihydroisoquinolin-3(4*H*)-one (10)

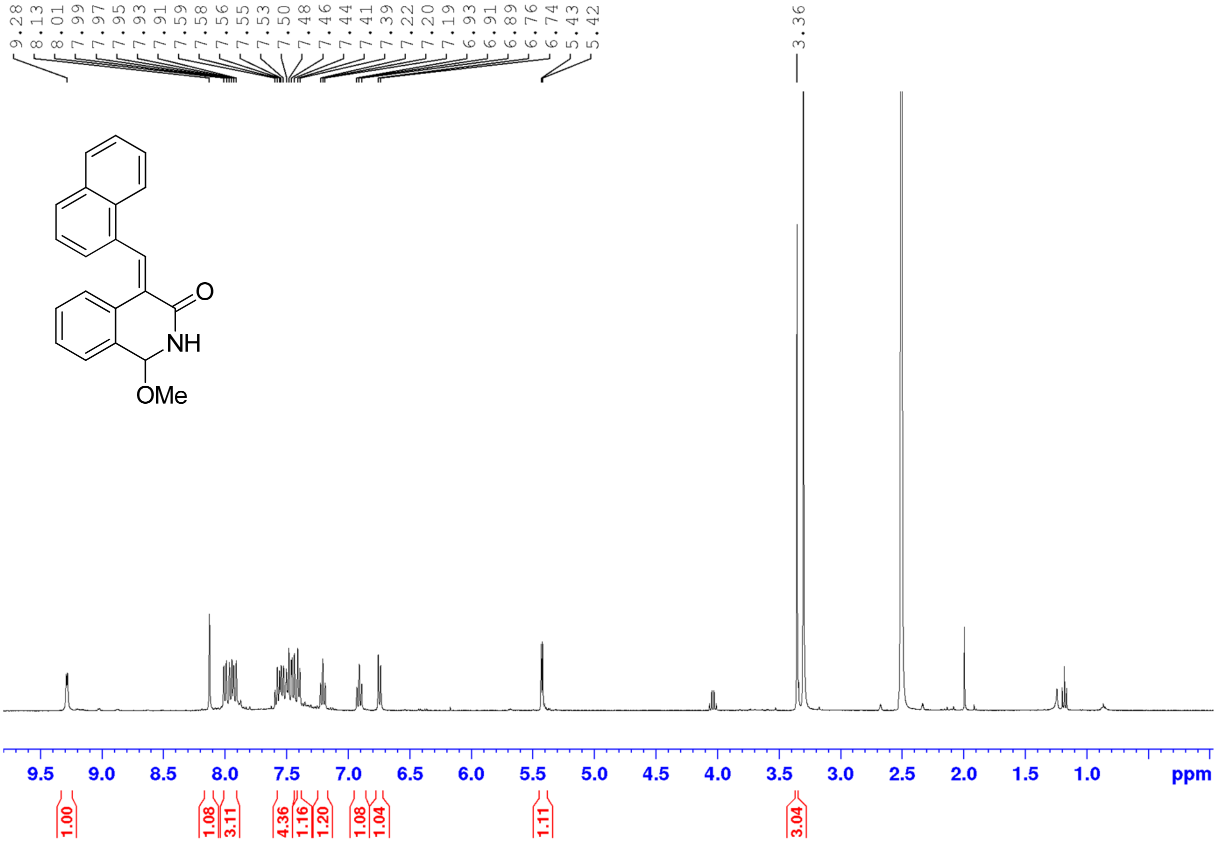

Figure S6. ^{1}H-NMR spectrum of **10**

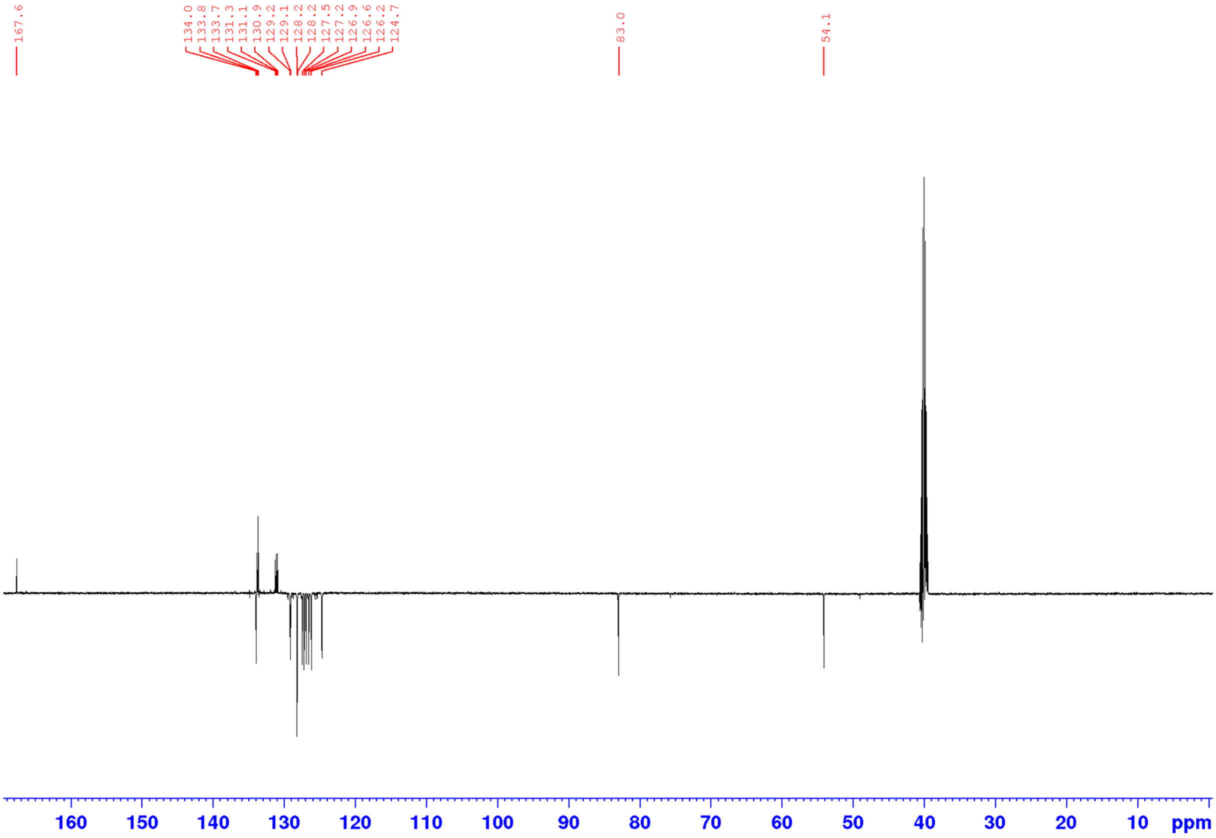

Figure S7. ^{13}C-NMR spectrum of **10**

3'-Hydroxy-1,2-dihydro-[1,4'-biisoquinolin]-3(4*H*)-one (11a)

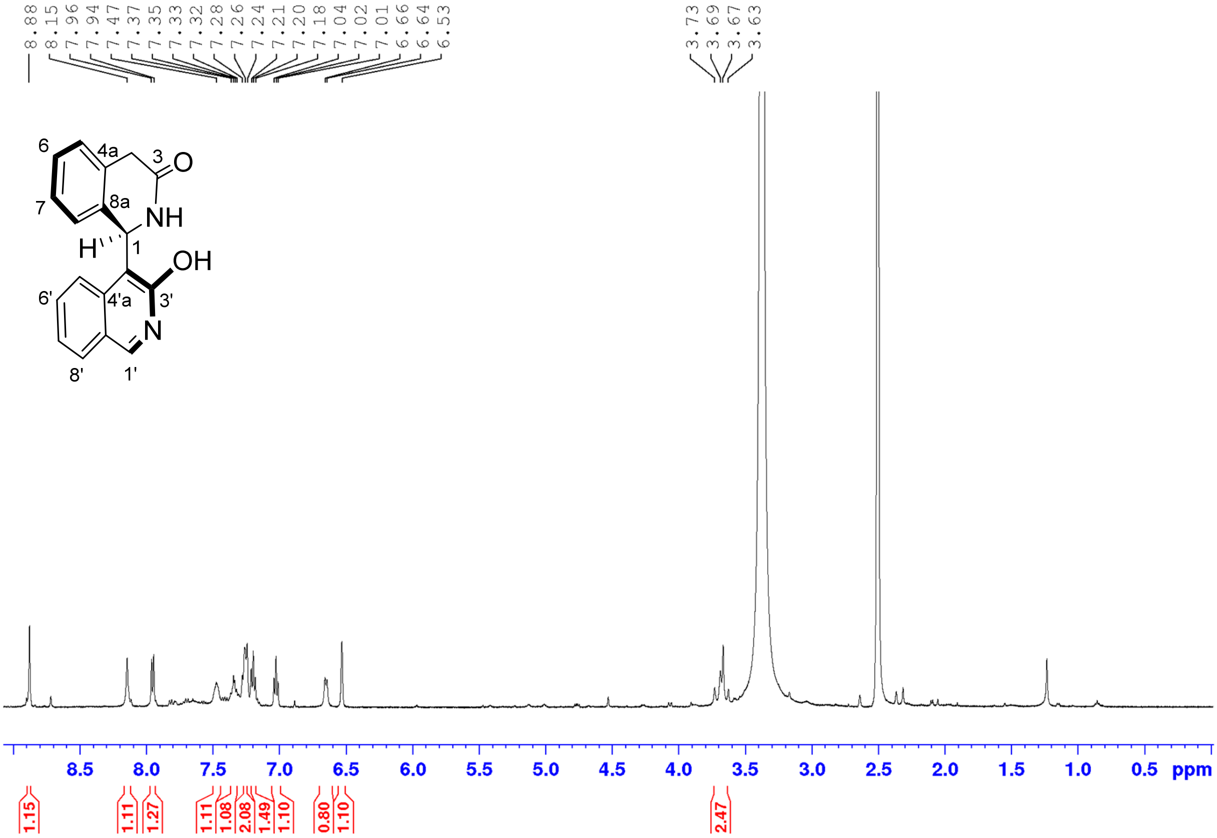

Figure S8. ^{1}H-NMR spectrum of **11a**

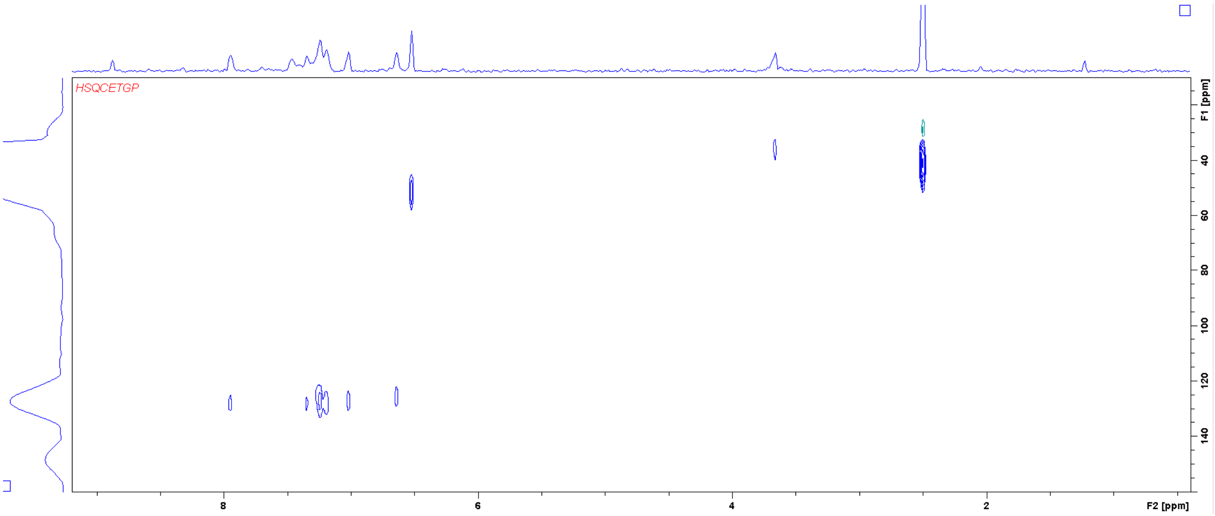

Figure S9. HSQC spectrum of **11a**

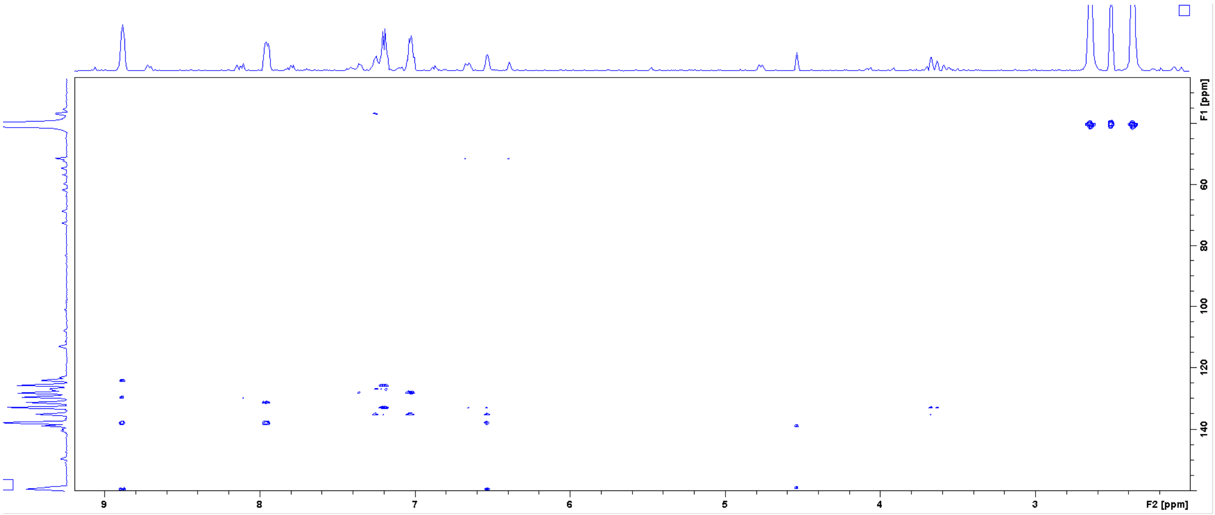

Figure S10. HMBC spectrum of **11a**

4-((Benzyl(methyl)amino)(phenyl)methyl)isoquinolin-3-ol (11b)

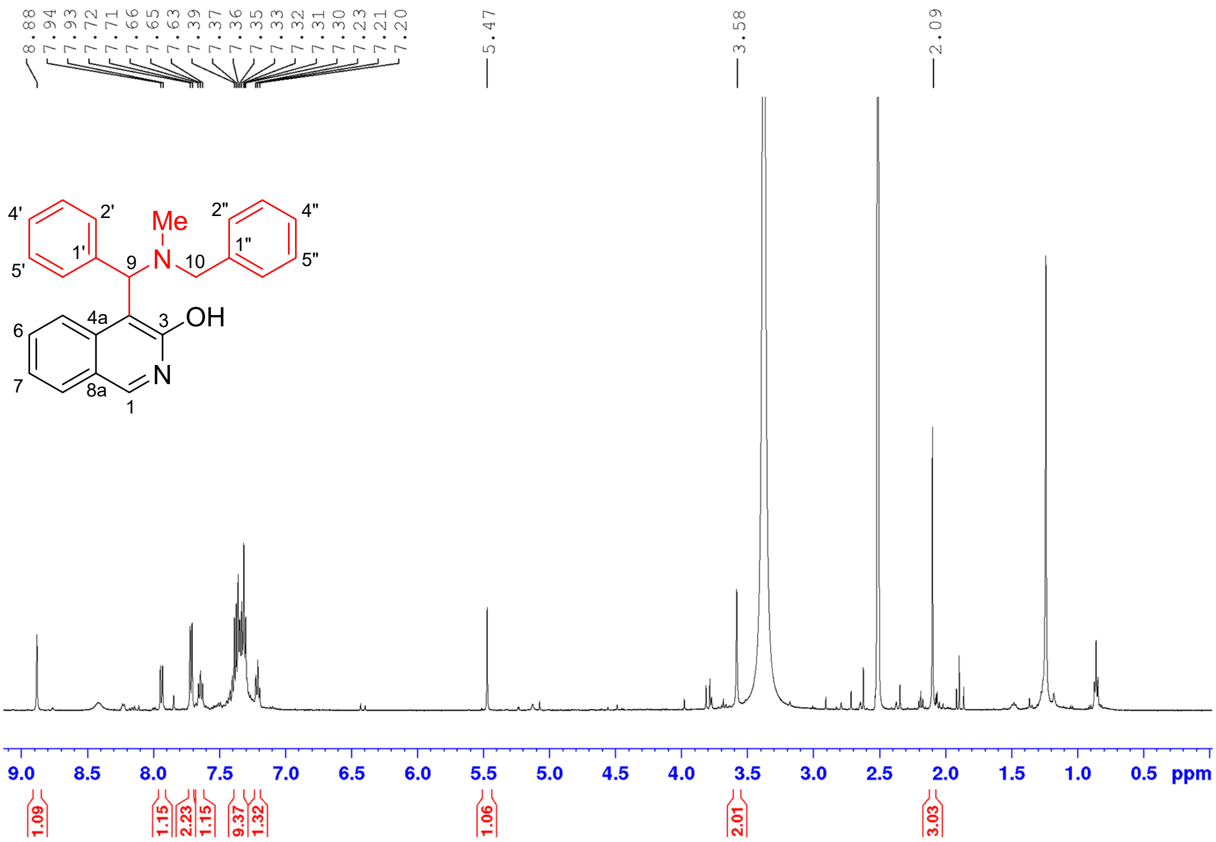

Figure S11. ^{1}H-NMR spectrum of 11**b**

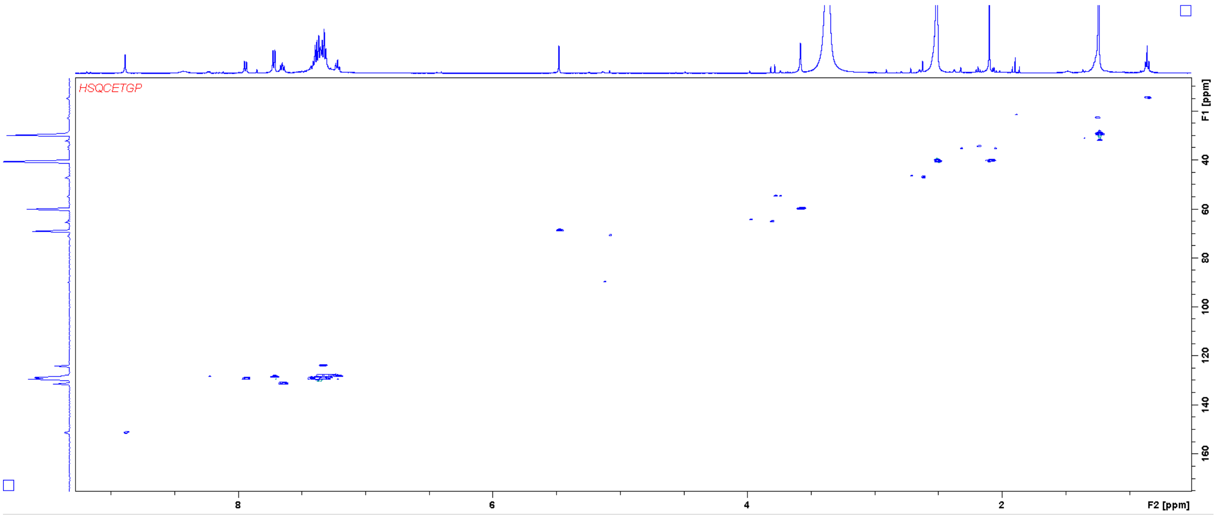

Figure S12. ^{13}C-NMR spectrum of **11b**

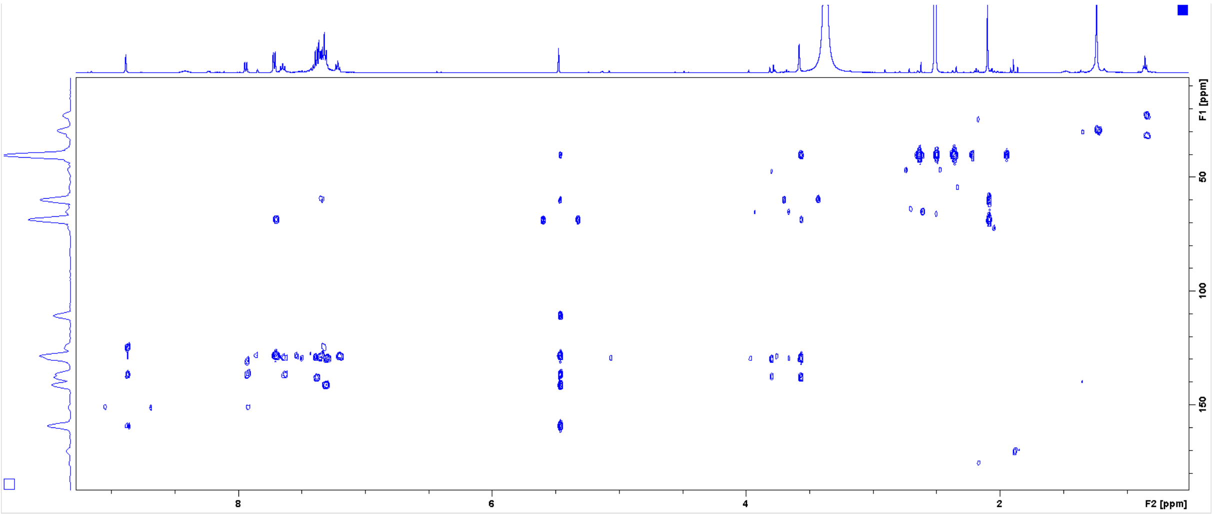

Figure S13. ^{13}C-NMR spectrum of **11b**

1-morpholino-1,2-dihydroisoquinolin-3(4*H*)-one (18)

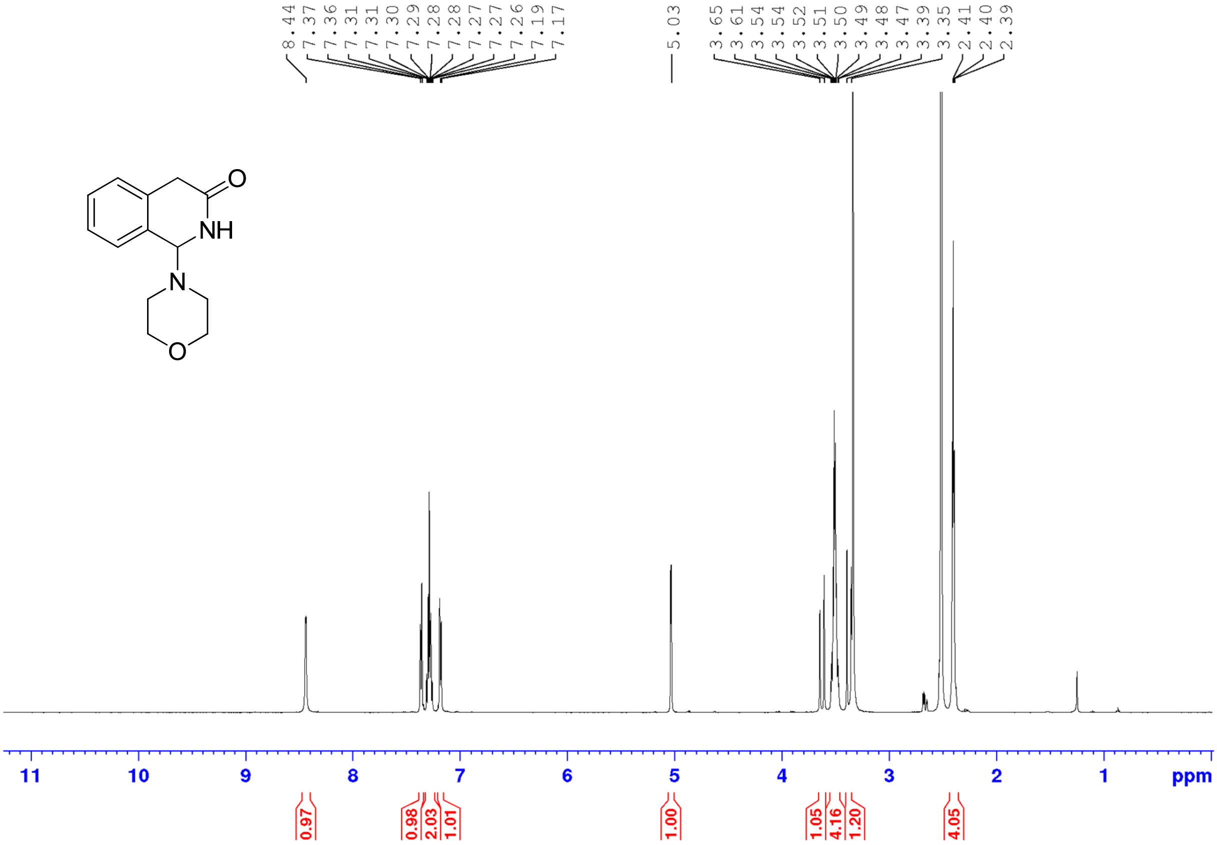

Figure S14. ^{1}H-NMR spectrum of **18**

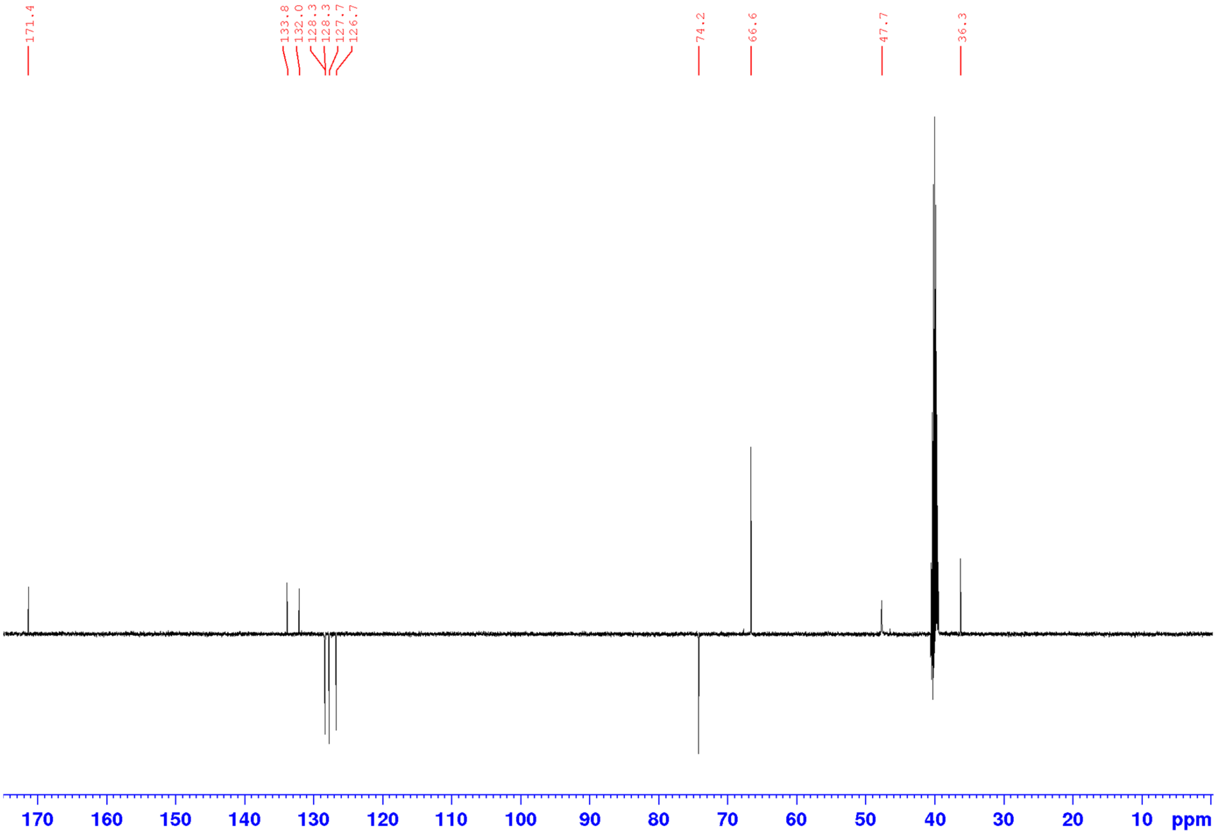

Figure S15. ^{13}C-NMR spectrum of **18**

(*E*)-1-morpholino-4-(naphthalen-1-ylmethylene)-1,2-dihydroisoquinolin-3(4*H*)-one (23aB)

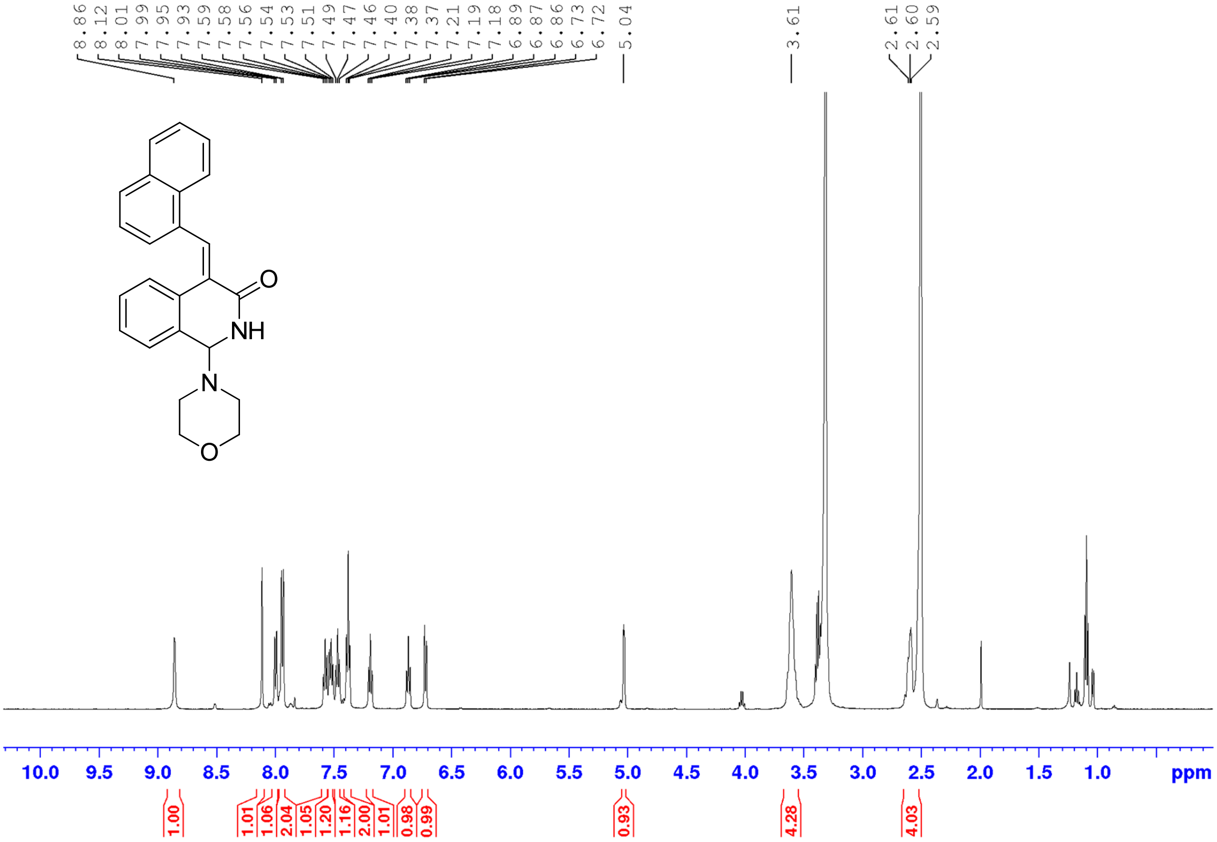

Figure S16. ^{1}H-NMR spectrum of **23aB**

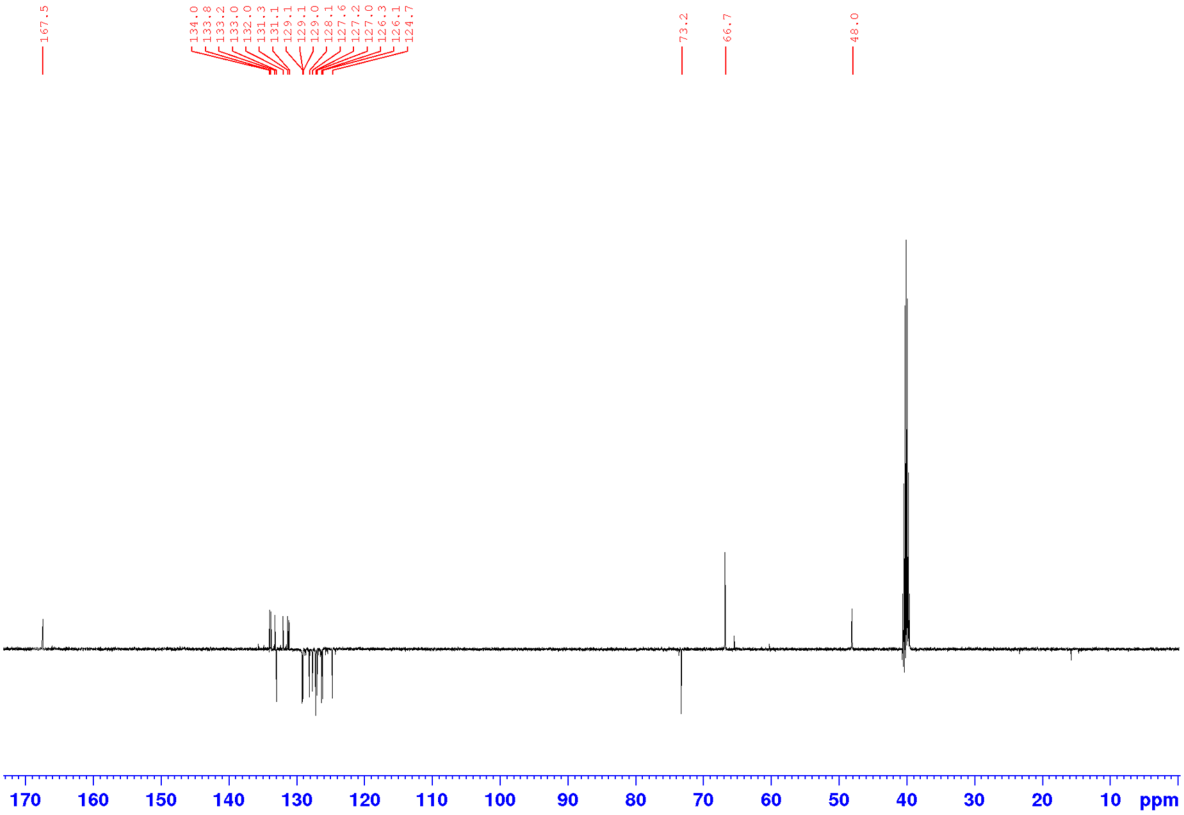

Figure S17. ^{13}C-NMR spectrum of **23aB**

4-(morpholino(phenyl)methyl)isoquinolin-3-ol (23bA)

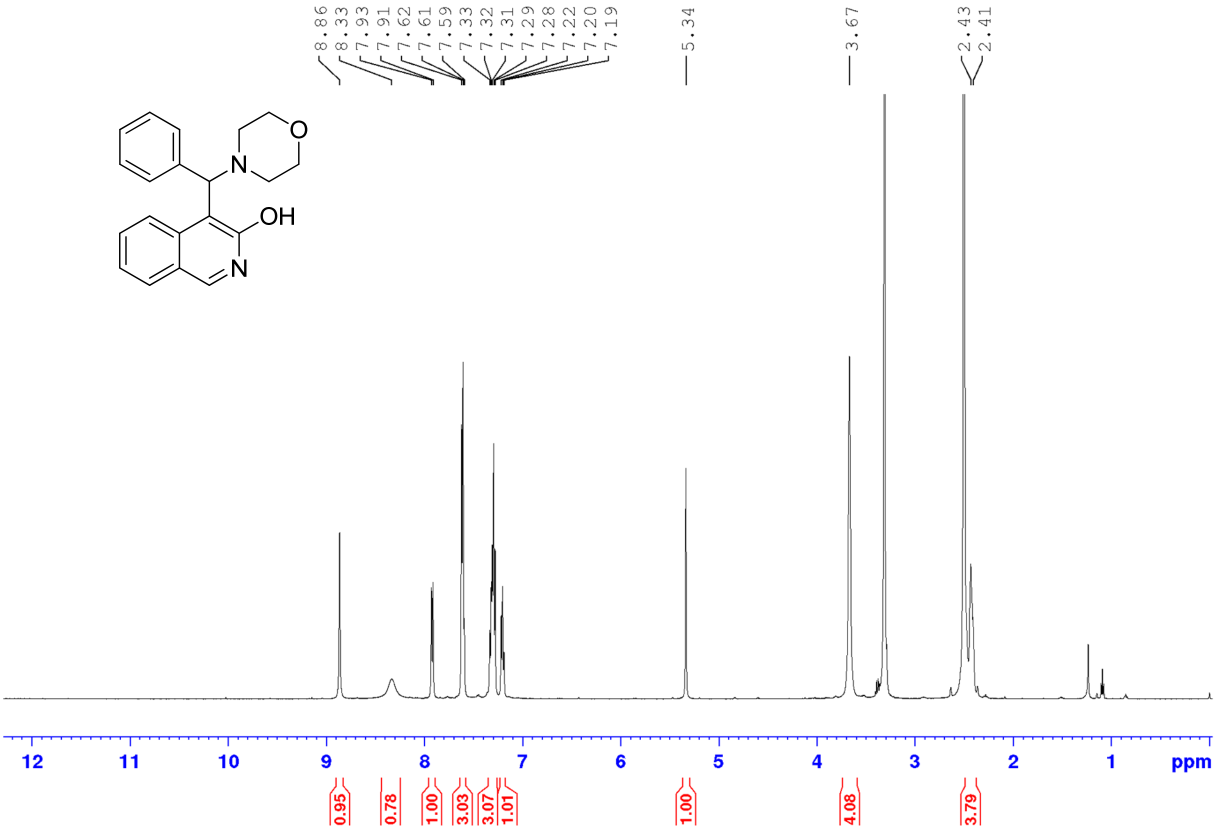

Figure S18. ^{1}H-NMR spectrum of **23bA**

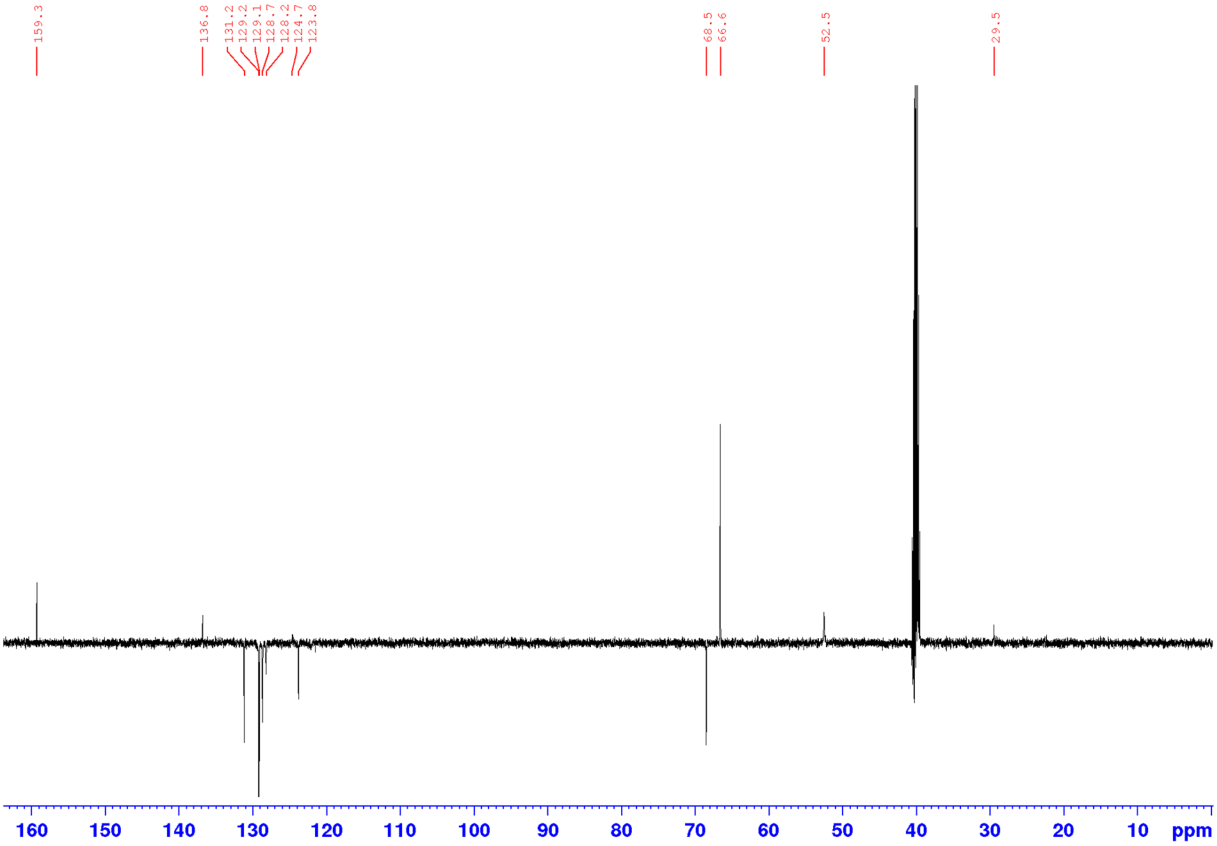

Figure S19. ^{13}C-NMR spectrum of **23bA**
